# Supplementary material for: Comparison of the Accuracy of Epistasis and Haplotype Models for Genomic Prediction of Seven Human Phenotypes
Source: Biomolecules. 2023 Oct 3;13(10):1478. doi: 10.3390/biom13101478 (PMC10604971; doi:10.3390/biom13101478)
Supplement: Supplementary file 1 [file biomolecules-13-01478-s001.zip › biomolecules-2574014-supplementary.pdf]

Supplementary Material

Comparison of the Accuracy of Epistasis and Haplotype Models for Genomic Prediction of Seven Human Phenotypes

Zuoxiang Liang, Dzianis Prakapenka, Yang Da

Department of Animal Science, University of Minnesota, Saint Paul, MN 55108, USA

Table S1. Prediction accuracy of alternative models for weight (WT) from 10-fold validations.

Prediction model	Observed prediction accuracy	Accuracy increase (%)
A+D	0.323	0%
A	0.322	-0.31%
A+D+AA	0.324	0.31%
A+D+AA+AD	0.325	0.62%
A+D+AA+DD	0.325	0.62%
A+D+AA+AD+DD	0.325	0.62%
**A+D+AD**	0.325	0.62%
A+D+DD	0.325	0.62%
A+D+AA+AD+DD+AAA+AAD	0.325	0.62%

The model in bold face is the best prediction model with the highest prediction accuracy and smallest number of effect types. A is additive values. D is dominance values. A×A, A×D and D×D are second-order (pairwise) epistasis values. A×A×A, A×A×D, A×D×D and D×D×D are third-order epistasis values. A×A×A×A, A×A×A×D, A×A×D×D, A×D×D×D and D×D×D×D are fourth-order epistasis values. Entries in italic are heritability estimates greater than 0.01 for the initial prediction models.

Table S2. Prediction accuracy of alternative models for body mass index (BMIo) from 10-fold validations.

Prediction model	Observed prediction accuracy	Accuracy increase (%)
A+D	0.322	0%
A	0.322	0%
A+D+AA	0.322	0%
A+AA+AD+DD	0.324	0.62%
A+AD+DD	0.325	0.93%
**A+AD**	0.325	0.93%
A+D+AA+AD+DD	0.324	0.62%

The model in bold face is the best prediction model with the highest prediction accuracy and smallest number of effect types. A is SNP additive values. D is SNP dominance values. H is haplotype additive values. AA is A×A values. AD is A×D values. DD is D×D values.

Table S3. Prediction accuracy of alternative models for high density lipoproteins (HDL) from 10-fold validations.

Prediction model	Observed prediction accuracy	Accuracy increase (%)
A+D	0.290	0%
A	0.287	-1.03%
A+D+AA	0.295	1.72%
**A+D+AA+AD**	0.297	2.41%

The model in bold face is the best prediction model with the highest prediction accuracy and smallest number of effect types. A is SNP additive values. D is SNP dominance values. H is haplotype additive values. AA is A×A values. AD is A×D values.

Table S4. Genomic heritability estimates with standard deviations from 10-fold validations.

	HDL	LDL	TC	TG	HTo	WT	BMIo
				SNP model (A+D)			
A	0.386 ± 0.009	0.408 ± 0.020	0.389 ± 0.011	0.260 ± 0.010	0.740 ± 0.010	0.472 ± 0.010	0.415 ± 0.011
D	0.124 ± 0.018	0.177 ± 0.028	0.104 ± 0.012	0.124 ± 0.038	0.202 ± 0.015	0.093 ± 0.012	0.046 ± 0.022
Total heritability	0.510 ± 0.014	0.585 ± 0.026	0.493 ± 0.016	0.383 ± 0.035	0.942 ± 0.014	0.565 ± 0.015	0.462 ± 0.017
				Epistasis model			
Prediction model	A+D+AA+AD	A+D+AA	A+D+AA	A+D+AA	A+D+AA	A+D+AD	A+AD
A	0.243 ± 0.012	0.286 ± 0.023	0.327 ± 0.012	0.211 ± 0.037	0.654 ± 0.009	0.460 ± 0.011	0.398 ± 0.013
D	0.056 ± 0.024	0.037 ± 0.023	0.087 ± 0.013	0.069 ± 0.045	0.137 ± 0.014	0.064 ± 0.019	-
A × A	0.358 ± 0.035	0.429 ± 0.063	0.162 ± 0.043	0.171 ± 0.134	0.183 ± 0.013	-	-
A × D	0.115 ± 0.051	-	-	-	-	0.188 ± 0.056	0.265 ± 0.08
Total heritability	0.771 ± 0.029	0.752 ± 0.047	0.576 ± 0.035	0.451 ± 0.083	0.975 ± 0.006	0.712 ± 0.036	0.663 ± 0.071
				Integrated model with epistasis and haplotype effects			
Prediction model	A+D+AA+AD+H	A+D+AA+H	A+D+AA+H	A+D+AA+H	A+D+AA+H	A+D+AD+H	A+AD+H
A	0.102 ± 0.022	0.010 ± 0.007	0.010 ± 0.007	0.072 ± 0.041	0.361 ± 0.016	0.010 ± 0.007	0.119 ± 0.022
D	0.041 ± 0.023	0.04 ± 0.024	0.078 ± 0.013	0.062 ± 0.038	0.145 ± 0.012	0.043 ± 0.017	-
A × A	0.042 ± 0.021	0.134 ± 0.065	0.017 ± 0.011	0.082 ± 0.096	0.001 ± 0.001	-	-
A × D	0.264 ± 0.05	-	-	-	-	0.105 ± 0.04	0.135 ± 0.078
H	0.332 ± 0.034	0.517 ± 0.049	0.403 ± 0.026	0.206 ± 0.044	0.494 ± 0.024	0.427 ± 0.039	0.356 ± 0.019
Total heritability	0.78 ± 0.032	0.702 ± 0.037	0.538 ± 0.021	0.421 ± 0.07	1 ± 0	0.687 ± 0.028	0.61 ± 0.071

A is SNP additive values. D is SNP dominance values. H is haplotype additive values. AA is A×A values. AD is A×D values. HDL is the normality transformed high density lipoproteins. LDL is the normality transformed low density lipoproteins. TC is the normality transformed total cholesterol. TG is the normality transformed triglycerides. WT is the normality transformed weight (WT). HTo is the original phenotypic observations without normality transformation of height, BMIo is the original phenotypic observations without normality transformation of body mass index.
